# Editorial: Molecular landscapes of human papillomavirus-related squamous cell carcinoma

**DOI:** 10.3389/fonc.2023.1215142

**Published:** 2023-05-31

**Authors:** Yujie Shen, Jinhui Liu

**Affiliations:** ^1^ Department of Otorhinolaryngology, Eye & ENT Hospital of Fudan University, Shanghai, China; ^2^ Department of Gynecology, The First Affiliated Hospital of Nanjing Medical University, Nanjing, Jiangsu, China

**Keywords:** data mining, human papillomavirus, squamous cell carcinoma, biomarker, prognostic

Human papillomavirus (HPV) infection is among the leading etiologies of various malignancies (1), notably squamous cell carcinoma (SCC) which can arise at multiple anatomical sites including the cervix, anus, oral cavity, and oropharynx (2–5). Despite considerable progress in the prevention and management of SCC, it remains a highly lethal malignancy. Therefore, the exploration of novel methodologies and techniques to enhance early detection, prevention, and therapeutic outcomes for SCC has emerged as a pressing mandate.

As an emerging technological tool, data mining has been widely applied in the field of medicine. It can assist researchers in extracting valuable information from massive data, such as identifying the pathogenesis of cancer (6), recognizing patients’ risk factors (7), and predicting prognosis (8). Additionally, data mining can help discover novel treatment strategies and drugs, as well as optimize existing treatment regimens. In terms of SCC, Gao et al. evaluated the expression of *KPNA2*, its signaling pathways, and prognostic relevance in HPV^+^ tongue squamous cell carcinoma via data mining. In cervical cancer, Feng et al. investigated the association between single nucleotide polymorphisms of different genes (*EXOC1*, *BCL2*, *CCAT2*, and *CARD8*) and susceptibility to cervical cancer using multiplex PCR combined with next-generation sequencing. In head and neck cancer, researchers integrated bioinformatics analysis and found that the expression of *MDM2* mRNA and protein was significantly higher in HPV^+^ HNSCC than in HPV^-^ HNSCC patients, suggesting that overexpression of *MDM2* may interfere with the p53-related pathway and thus affect the occurrence and proliferation of HPV-related HNSCC (Bouzid et al.).

Despite these research papers published in this Research Topic can provide unique insights into HPV-related squamous cell carcinoma, research on HPV-related tumors faces many challenges and difficulties. Firstly, the relationship between HPV infection and tumor development is very complex, and its pathogenic mechanisms have not been fully elucidated. Secondly, the clinical features and prognosis of HPV-related tumors are related to factors such as HPV viral type, tissue type, and immune status, making research difficult and heterogeneous. In addition, the diagnosis, treatment, and prevention of HPV-related tumors also face many challenges, such as standardization and uniformity of HPV virus detection (9), individualization and precision of treatment plans, etc. Therefore, strengthening research on HPV-related tumors, exploring their pathological and physiological mechanisms, and developing more effective prevention and treatment strategies are of great significance for safeguarding public health and human health.

## Author contributions

YS and JL wrote the manuscript. YS and JL edited the language. All authors contributed to the article and approved the submitted version.
